# Draft Genome Sequence of the Rifamycin Producer *Amycolatopsis rifamycinica* DSM 46095

**DOI:** 10.1128/genomeA.00662-14

**Published:** 2014-07-03

**Authors:** Anjali Saxena, Rashmi Kumari, Udita Mukherjee, Priya Singh, Rup Lal

**Affiliations:** Department of Zoology, University of Delhi, Delhi, India

## Abstract

*Amycolatopsis rifamycinica* DSM 46095 is an actinobacterium that produces rifamycin SV, an antibiotic used against *Mycobacterium tuberculosis*. Here, we present the draft genome of DSM 46095, which harbors a novel rifamycin polyketide biosynthetic gene cluster (*rif* PKS) that differed by 10% in nucleotide sequence from the already reported *rif* PKS cluster of *Amycolatopsis mediterranei* S699.

## GENOME ANNOUNCEMENT

*Amycolatopsis mediterranei* DSM 46095 was first isolated from a soil sample in an arid region near Alice Springs, Australia (1). At the time of isolation, this strain was named as *Nocardia mediterranei* (1), then *Amycolatopsis mediterranei* (2), and finally *Amycolatopsis rifamycinica* (3). *A. rifamycinica* DSM 46095 was reported to produce rifamycin SV (1), which is a semisynthetic derivative of rifamycin B and is used for curing tuberculosis.

The rifamycin polyketide synthase (*rif* PKS) gene cluster, involved in the synthesis of rifamycin B, was characterized from two strains of *A. mediterranei*, S699 (4) and LBGA 3136 (5). While the *rif* PKS gene clusters were essentially identical in both of these strains (4–6), the partially characterized (~4 kb) *rif* PKS-like gene cluster from *A. rifamycinica* DSM 46095 showed 10% differences in nucleotide sequences compared to the *rif* PKS clusters from S699 and LBGA 3136 (7). To further understand the novel rifamycin polyketide synthase gene cluster, the genome sequencing of this strain was performed.

Total genomic DNA of strain 46095 was sequenced by the Illumina Genome Analyzer platform IIx using a PCR-free-based approach (paired-end library, 2 kb [*n* = 12,157,214] and 500 bp [*n* = 1,4035,608]). The draft genome sequence (9.20 Mb) of strain 46095 was assembled (150× coverage) into 88 contigs (>500 bp [±10 bp]) using the ABySS 1.3.5 assembler (8) set at a k-mer size of 63. The final validated assembly (N_*50*_ contigs, 491,147 bp) was annotated using RAST version 4.0 (9) and the NCBI Prokaryotic Genomes Annotation Pipeline (PGAP) (http://www.ncbi.nlm.nih.gov/genomes/static/Pipeline.html). There were 8,307 coding sequences (CDSs), with an average GC content of 71.8%, 414 subsystems, and 83 pseudogenes. These CDSs fall into 6,879 functional clusters of orthologous groups (COGs) with the presence of 2,491 hypothetical proteins. By using the antiSMASH server (10), we observed ~82 secondary metabolite gene clusters encoding type I specific polyketide synthases (PKS), type II PKS, nonribosomal peptide synthetases (NRPS), hybrid PKS, lantipeptides, lycopenes, and terpenes in the draft genome.

The *rif* PKS gene cluster in strain 46095 was represented by a continuous fragment of ~85 kb on contig number 39 that contained *rifA*, *rifB*, *rifC*, *rifD*, and *rifE* open reading frames (ORFs). Acyltransferase (AT) and ketosynthase (KS) domains present on these ORFs showed 90% homology with that of *A. mediterranei* S699 (4). Apart from a unique *rif* PKS cluster, eight other PKS, seven NRPS, and three hybrid NRPS/PKS clusters were also present in the genome (11).

A complex regulatory network of 590 transcriptional regulators, including genes for 98 histidine kinases, 82 response regulators, and 1 phosphotransferase protein, was present. Fifty-six tRNAs, 18 rRNAs, 1,242 tandem repeats, and 2 clustered regularly interspaced short palindromic repeat (CRISPR) elements were also identified. Average nucleotide identity (ANI) (12) analysis revealed that the draft genome of *A. rifamycinica* 46095 is phylogenetically related to *Amycolatopsis mediterranei* S699 (92.55%) (6), *Amycolatopsis mediterranei* U32 (92.53%) (13), and *Amycolatopsis mediterranei* RB (92.52%) (CP003777).

The sequence information of a novel rifamycin biosynthetic gene cluster of *A. rifamycinica* 46095 will help in the analysis of the structure of polyketide being produced by this strain. This information, supplemented with other genomic information, might also be used to design combinatorial strategies like domain replacement and domain inactivation (14) to produce rifamycin analogs (15).

### Nucleotide sequence accession numbers.

This whole-genome shotgun project has been deposited at DDBJ/EMBL/GenBank under the accession number JMQI00000000. The version described in this paper is version JMQI01000000.
